# Gd-EOB-DTPA-enhanced magnetic resonance imaging combined with T1 mapping predicts the degree of differentiation in hepatocellular carcinoma

**DOI:** 10.1186/s12885-016-2607-4

**Published:** 2016-08-12

**Authors:** Zhenpeng Peng, Mengjie Jiang, Huasong Cai, Tao Chan, Zhi Dong, Yanji Luo, Zi-Ping Li, Shi-Ting Feng

**Affiliations:** 1 Department of Radiology, The First Affiliated Hospital, Sun Yat-Sen University, 58th, The Second Zhongshan Road, Guangzhou, Guangdong 510080 China; 2 Department of Radiology, Hospital of Stomatology, Guanghua School of Stomatology, Guangdong Provincial Key Laboratory of Stomatology, Sun Yat-Sen University, Guangzhou, 510055 China; 3 Medical Imaging Department, Union Hospital, Hong Kong, 18 Fu Kin Street, Tai Wai, Shatin, N.T Hong Kong

**Keywords:** Gd-EOB-DTPA, T1 mapping, Hepatocellular Carcinoma, Edmondson-Steiner grade, Differentiated degrees

## Abstract

**Background:**

Variable degrees of differentiation in hepatocellular carcinoma(HCC)under Edmondson-Steiner grading system has been proven to be an independent prognostic indicator for HCC. Up till now, there has been no effective radiological method that can reveal the degree of differentiation in HCC before surgery. This paper aims to evaluate the use of Gd-EOB-DTPA-Enhanced Magnetic Resonance Imaging combined with T1 mapping for the diagnosis of HCC and assessing its degree of differentiation.

**Methods:**

Forty-four patients with 53 pathologically proven HCC had undergone Gd-EOB-DTPA enhanced MRI with T1 mapping before surgery. Out of the 53 lesions,13 were grade I, 27 were gradeII, and 13 were grade III. The T1 values of each lesion were measured before and at 20 min after Gd-EOB-DTPA administration (T1p and T1e). The absolute reduction in T1 value (T1d) and the percentage reduction (T1d %) were calculated. The one-way ANOVA and Pearson correlation were used for comparisons between the T1 mapping values.

**Results:**

The T1d and T1d % of grade I, II and III of HCC was 660.5 ± 422.8ms、295.0 ± 99.6ms、276.2 ± 95.0ms and 54.0 ± 12.2 %、31.5 ± 6.9 %、27.7 ± 6.7 % respectively. The differences between grade Iand II, grade Iand III were statistically significant (*p* < 0.05), but there was no statically significant difference between grade II and III. The T1d % was the best marker for grading of HCC, with a Spearman correlation coefficient of −0.676.

**Conclusions:**

T1 mapping before and after Gd-EOB-DTPA administration can predict degree of differentiation in HCC.

## Background

Hepatocellular carcinoma (HCC) ranks as the sixth most common cancer worldwide and is responsible for about 9 % of the cancer-related deaths globally [1]. Many new developments in medical imaging help to improve detection, characterization, and prognostication of HCC, eg contrast-enhanced ultrasonography (CEUS), multidetector computer tomography (MDCT), diffusion weighted magnetic resonance imaging (DW-MRI), and Gd-EOB-DTPA enhanced MRI [1–4]. Gadoxetic acid is a hepatocyte-specific contrast agent with a pendant ethoxybenzyl group covalently attached to the extracellular contrast agent gadopentetate dimeglumine, which can be taken up by functioning hepatocytes. It is eliminated in equal quantities by the urinary and biliary systems [5–8]. Therefore, it enables the assessment of tumor vascularity as well as other hepatocellular-specific properties within the tumor. Thus Gd-EOB-DTPA has been proven valuable in detection and differential diagnosis of focal liver lesions, especially between early HCC and dysplastic nodule(DN) [3, 9]. As HCCs in different degree of differentiation retain variable degree of normal liver function, they could potentially be classified using Gd-EOB-DTPA [10, 11].

Measurement of the T1 relaxation time in a lesion before and after Gd-EOB-DTPA administration allows quantitative evaluation of Gd-EOB-DTPA uptake, which potentially reveals related properties of the lesion [12]. Unlike MR signal intensity, which is affected by many technical factors and variable MR imaging parameters, T1 relaxation time is a characteristic of the imaged tissue. T1 mapping can hence be used for quantitative measurements and characterization of tissues [4, 12, 13]. T1mapping on Gd-EOB-DTPA-enhanced MRI has been shown to be able to quantitatively distinguish hepatic hemangiomas from metastatic tumors [5]. T1 mapping on Gd-EOB-DTPA-enhanced MRI also showed significant differences in T1 relaxation time in hepatobiliary phase and its rate of decrease (Δ %) between normal liver, non-alcoholic fatty liver disease (NAFLD) and non-alcoholic steatohepatitis (NASH) [4]. This method has also been successfully used to estimate liver function [12, 14].

As of to date, there has been no attempt to assess the degree of differentiation of HCC by medical imaging methods.

It has been established that Edmondson-Steiner grade was an independent predictor for early recurrence in HCC. Furthermore, Edmondson-Steiner grade has been shown to be related to other markers of poor prognosis, including microvascular invasion [15–19], expression of Nemo-like kinase (NLK), Protein disulfide isomerase (PDI), p21-activated protein kinase (PAK) 6, chemokine receptor CCR9 [20–23].

Previous studies have shown that different grades of HCCs appear differently on both dynamic contrast-enhanced (DCE) magnetic resonance imaging (MRI) and hepatobiliary-phase MR images. Well-differentiated HCC tends to show signals closer to that of liver parenchyma and less conspicuous hypointensity Gd-EOB-DTPA on hepatobiliary-phase images [10, 11, 24]. One study also indicated that maximum sensitivity of Gd-EOB-DTPA enhanced MRI was only 69 % even when diagnostic criteria including all previously reported pattern of HCC were adopted, missing some well-differentiated and moderately differentiated HCC [25]. It is noteworthy that the above studies were based on qualitative assessment. We propose the use of T1 mapping index on Gd-EOB-DTPA enhanced MRI that might predict Edmondson-Steiner grade of HCC, and hence improve the prognostication of HCC prior to surgery.

## Methods

### Study Population

This is a retrospective study conducted in accordance with ethical guidelines for human research and was compliant with the Health Insurance Portability and Accountability Act (HIPAA). As such, the study received IRB or ethical committee approval, and the requirement for informed consent was waived.

Between July 2012 and February 2015, 44 patients (41 males and 3 females) suffering from HCC who had undergone gadoxetic acid-enhanced MRI with a 3.0 Tesla (T) system within a week before hepatectomy were included. (Patients who were non-evaluable or with poor images were excluded.) Pathological diagnosis and grading were made according to Edmondson-Steiner grading system, see Table 1 [16, 19].

**Table 1:** Edmondson-Steiner grade

Edmondson-Steiner grade	
I	Cells with abundant cytoplasm and minimal nuclear irregularity
II	Greater nuclear irregularity and prominent nucleoli
III	Increased nuclear pleomorphism and angulation of the nuclei; Tumour giant cells were also more commonly seen
IV^a^	Poorly differentiated with marked nuclear pleomorphism, hyperchromatism and anaplasia

^a^Limited by study population, this study did not include HCC of Edmondson-Steiner grade IV

### MR imaging protocol

All MRI examinations were conducted with a clinical 3T whole body system (Magnetom Verio, Siemens Healthcare Sector, Erlangen, Germany). The body array coil (3T; 8-channel body matrix coil) was used in all examinations. All patients fasted for 6 ~ 8 h prior to examination and were trained for holding breath. Bellyband was used during examination. The sequences employed are listed in Table 2.

**Table 2:** MRI sequences used in the study

Sequence	TR(ms)	TE(ms)	FA	Scan time (s)	Slice thickness (mm)	Matrix	FOV
Plain scan							
T1WI	225	2.2	70	19.42	6	200 × 320	258 × 330
T2WI	2000	91	150	106	6	410 × 512	350 × 350
VIBE-T1mapping	4.4	1.2	2,12	20.15	2	154 × 256	248 × 330
DCE							
VIBE	3.3	1.2	13	8.21/phase	2	96 × 256	248 × 330
Hepatobiliary-phase							
T1WI	225	2.2	70	19.42	6	200 × 320	258 × 330
VIBE-T1mapping	4.4	1.2	2,12	20.15	2	154 × 256	248 × 330

All patients received a body weight adjusted dose of Gd-EOB-DTPA (Primovist ®, 0.1ml/kg body weight) administered as bolus injection with a flow rate of 1ml/s, flushed with 30 ml of normal saline at the same rate. The T1 relaxation times were measured on both non-contrast images and images obtained at 20mins after injection of the Gd-EOB-DTPA.

### Image analysis

**Fig. 1:**
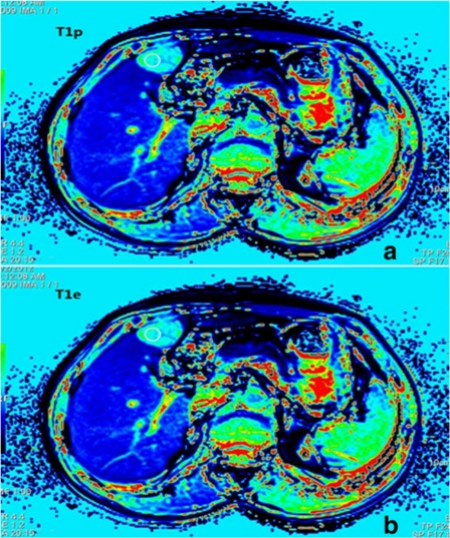
**a**, **b** T1 values in T1 mapping images was measured before (T1p) and after (T1e) administration of the contrast medium. The two ROIs were chosen at the same place in the same lesions

The T1 maps of the liver were generated with the evaluation tool for calculating T1 relaxation times (Siemens Leonardo Syngo 2009B). T1 values on T1 mapping images were measured before and after the administration of the contrast medium (recorded as T1p and T1e respectively) as shown in Fig. 1. Regions of interest (ROIs) were drawn in the most homogeneous appearing portion of the lesion, avoiding tumor capsule, necrosis, fat and vessels. Round ROIs were drawn as large as possible within the boundary of the lesion, and that the ROIs were identical in size and shape on images before and after contrast.

Two experienced radiologists drew the ROIs independently, and would reach a consensus by consultation if there were any conflict. Each lesion was measured for 3 times and the mean of 3 values was applied for calculating reduction of T1 values (T1d) after enhancement and its reduction ratio (T1d %) as follows:

$$ \mathrm{T}1\mathrm{d} = \mathrm{T}1\mathrm{p}-\mathrm{T}1\mathrm{e} $$

$$ \mathrm{T}1\mathrm{d}\% = \left[\left(\mathrm{T}1\mathrm{p}-\mathrm{T}1\mathrm{e}\right)/\mathrm{T}1\mathrm{p}\right]\mathrm{x}100\% $$

[8, 13]

T1 maps were also color-coded using a visualization tool of the open source OsiriX imaging software.

### Statistical analysis

All statistical analyses were done using SPSS (version 19.0, Statistical Package for Social Science,Chicago,USA). A one-way analysis of variance (ANOVA) was used to analyze differences of these values between different Edmondson-Steiner grades. Spearman correlation was also done between T1 mapping characteristics and different Edmondson-Steiner grades.

All statistical tests were two-sided and *p* value < 0.05 indicated significant difference.

## Results

The median age of patients was 54.9 (range 37–77). Altogether, 53 lesions in 44 patients confirmed and graded by pathology were analyzed in the study.

### Size

The lesion size of different grades showed no statistical significance, as shown in Table 3.

**Table 3:** Size of lesions for different Edmondson-Steiner grade

Edmondson-Steiner grade	No.	Diameters (mean, mm)^a^
I	13	31.3 ± 23.7
II	27	25.4 ± 23.5
III	13	38.8 ± 35.6
Total	53	30.1 ± 28.5

^a^There was no statistically significant difference between three groups (*P* = 0.389)

### Comparison of T1mapping for HCC in different Edmondson-Steiner grade

**Fig. 2:**
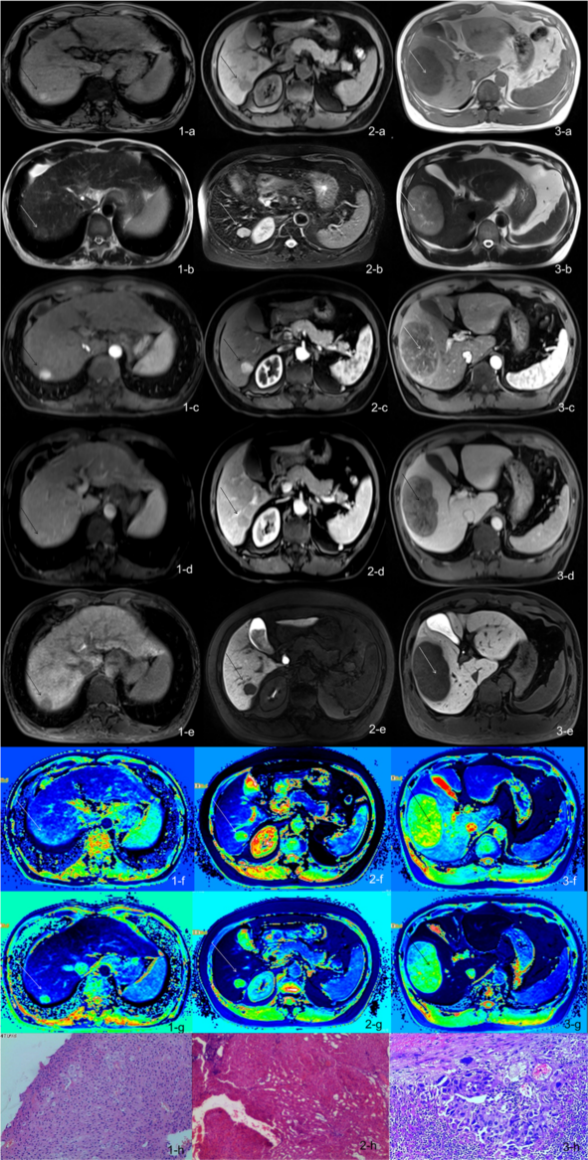
Row **a**: T1 weightedimages, Row **b**: T2 weightedI image, Row **c**: arterial phase image, Row **d**: potal venous phase image, Row **e**: hepatobiliary phase images; Row **f**: and Row **g**: T1 mapping of T1WI and hepatobiliary phase images; row **h**: corresponding pathological pictures. 1**a-h**, HCC (Edmondson-Steiner grade I). Lesion appeared hyperintense on routine sequences and hypointense in hepatobiliary-phase; T1p 892ms, T1e 388 ms, T1d = 504 ms, T1d % = 56.50 %; cells showed abundant cytoplasm and minimal nuclear irregularity. 2**a-h**, HCC (Edmondson-Steiner grade II), hypointense on T1WI and hepatobiliary phase images, “wash in and wash out”; T1p 1696 ms, T1e 1444 ms, T1d = 252 ms, T1d % = 14.90 %. 3**a-h**, HCC (Edmondson-Steiner grade III), hyperintense on T2WI images, hypointense on T1WI and hepatobiliary phase images; T1p 2134 ms, T1e 1494 ms,T1d = 640 ms,T1d % = 30.00 %. 2-h and 3-h, nuclear pleomorphism increased with increase in Edmondson-Steiner grade

**Fig. 3:**
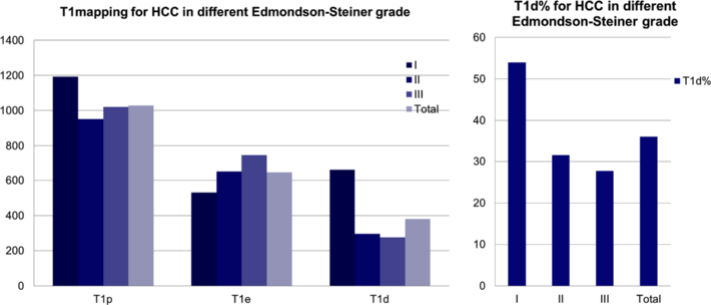
Values of T1p, T1e, T1d, T1d % in different groups and all of the studied lesions

HCC showed either hyperintensity or hypointensity on T1WI, and hypointensity on hepatobiliary-phase images. The higher the Edmondson-Steiner grade, the more conspicuous the difference between lesion and normal liver parenchyma (Fig. 2). T1p, T1e, T1d and T1d % were measured and calculated, the results are shown in Fig. 3. There was no statistical significance of T1p and T1e between three groups (*p* = 0.144, 0.059). But both T1d and T1d % showed statistical differences between the groups. T1d ranged from 141ms to 1913ms, and T1d % from 14.8 to 74.8 %. In general, T1d and T1d % decreased with increase in Edmondson-Steiner grade.

**Fig. 4:**
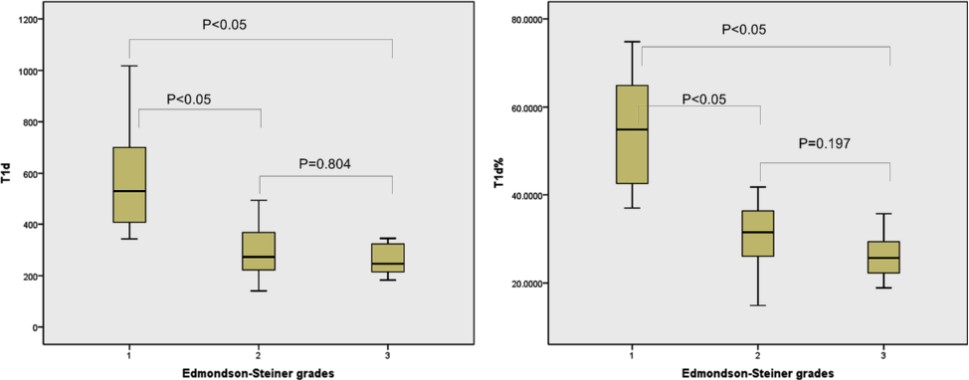
The average value of T1d and T1d % in HCC of different Edmondson-Steiner grades. Box-and-whisker plots showed that there was statistical significance of T1d and T1d % between grade I and the other two groups, but no statistically significant difference between grade II and grade III lesions

Mutivariate analysis showed there was statistical significance of T1d and T1d % between both grade I-grade II and grade I-grade III (*p* < 0.05). However the data from group II and group III were not different from one another (*p* = 0.804,0.197) (Fig. 4).

### Correlation analysis

Spearman Correlation analysis manifested positive correlation between T1e and Edmondson-Steiner grade (*P* < 0.05), as well as negative correlations between T1d and Edmondson-Steiner grade, T1d % and Edmondson-Steiner grade (*P* < 0.05). Of the above, T1d % had the best correlation with a correlation index 0.676 (Table 4).

**Table 4:** Correlation analysis between T1p, T1e, T1d, T1d % and Edmondson-Steiner grade

	T1p	T1e	T1d	T1d %
Correlation index	−0.065	0.335	−0.570	−0.676^a^
P	0.643	0.014	0.000	0.000
N	53	53	53	53

^a^T1d % had the best correlation with Edmondson-Steiner grade with a correlation index 0.676

## Discussion

This study showed that HCC of Edmondson-Steiner grade I could be distinguished from grade II and III lesions using T1d and T1d %. The higher the grade, the lower the T1d and T1d %. From the above, it was suggested that uptake of Gd-EOB-DTPA decreases with increase in Edmondson-Steiner grade. Generally, 5–10 % of HCC are Edmondson-Steiner grade I lesions. But in this study, grade I HCC accounted for 24.5 % (13/53) of the samples, which could be due to the higher proportion of small HCC in the sample [26].

Gd-EOB-DTPA has been widely used in the HCC evaluation and proved to be helpful for its diagnosis, differential diagnosis and grading. Some previous studies found that hypointensity on gadoxetic acid–enhanced hepatobiliary phase images and hyperintensity on high-b-value DWI suggest well-differentiated HCCs rather than benign hepatocellular nodules, and might predict worse histological grades of HCC [27, 28]. Schelhorn et al. [29] also reported that the enhancement patterns changed with different grades of HCC. On the other hand, it has been shown that 50 % of well-differentiated HCCs exhibit isointensity or hyperintensity in the hepatobiliary-phase, causing difficulty in imaging diagnosis, especially in patients with worse Child-Pugh class [10, 11, 24, 25, 30]. It is known that cell morphology of well-differentiated HCC is similar to that of normal liver cell. The ultra microstructures of well-differentiated HCC cells as revealed by electron microscope also exhibit similarities in cell nucleus, organelle, contact of cell, etc. to that normal liver cell. It is therefore believed that well-differentiated HCC could have preserved liver cell functions [31], which has also been suggested in an imaging study on animal HCC model [32]. Uptake of Gd-EOB-DTPA in hepatobiliary phase in HCC is determined by expression of OATP1B3, which expression varies in HCC of different grades [33]. However, the minor difference between HCC of different grades, as well as between well-differentiated HCC and normal liver tissue, could be difficult to appreciate by qualitative assessment.

This study confirmed that quantitative analysis based on T1 mapping allowed differentiation between different grades of HCC. Of the different quantitative measurements studied, T1d % had the best correlation with histological grades. The results showed that T1d % for grade I lesions was 54.0 ± 12.2 %, and that for grade II and III lesions were 31.5 ± 6.9 and 27.7 ± 6.7 % respectively. Therefore T1d % of higher than 50 % would suggest that an HCC is likely to be of Edmondson-Steiner grade I. Theoretically, T1p and T1e, could also be different in different Edmondson-Steiner grades; however, this was not supported by the results. It is suspected that this could in part be related to abnormal blood perfusion due to artery-to-vein or artery-to-portal vein shunting [34], which would reduce the T1 shortening effect of Gd-EOB-DTPA.

Previous studies have suggested that Edmondson-Steiner grade is an independent factor affecting prognosis/recurrence of HCC [15–20], which should be taken into account when considering therapeutic strategy [16, 19]. In addition, liver cells take up Gd-EOB-DTPA via the receptors OATP1B1, OATP1B3 and NTCP. OATP1B3 is also the transport for some anti-cancer drugs [32, 35–39]. Therefore, tumor behavior in terms of Gd-EOB-DTPA uptake might also predict effect of therapy. The results obtained by this study held promise to predict tumor grading patients prior to surgery, which might also help to choose between treatment options.

There are limitations in the current study. Firstly, the retrospective nature of the study could not avoid sampling bias. Secondly, due to the relatively small number of lesions, a model that describes the relations between the best indicator of T1d % and the individual Edmondson-Steiner grades could not be formulated.

## Conclusion

T1 mapping before and after Gd-EOB-DTPA administration can help to classify the HCC in terms of Edmondson-Steiner grade. The percentage reduction of T1 value after contrast in hepatobiliary phase was the best indication for such classification of HCC.

## Abbreviations

CEUS, contrast-enhanced ultrasonography; DCE-MRI, dynamic contrast-enhanced magnetic resonance imaging; DW-MRI, diffusion weighted magnetic resonance imaging; HCC, hepatocellular carcinoma; MDCT, multidetector computer tomography; NAFLD, non-alcoholic fatty liver disease; NASH, non-alcoholic steatohepatitis; NLK, Nemo-like kinase; PAK6, p21-activated protein kinase6; PDI, protein disulfide isomerase
